# Association of DNA repair genes polymorphisms with childhood acute lymphoblastic leukemia: a high-resolution melting analysis

**DOI:** 10.1186/s13104-022-05918-3

**Published:** 2022-02-14

**Authors:** Shahrzad Zehtab, Mahla Sattarzadeh Bardsiri, Roohollah Mirzaee Khalilabadi, Mohsen Ehsan, Ahmad Fatemi

**Affiliations:** 1 Department of Hematology and Medical Laboratory Sciences, Faculty of Allied Medicine, Kerman University of Medical Sciences, Kerman, Iran; 2 Cell Therapy and Regenerative Medicine Comprehensive Center, Kerman University of Medical Sciences, Kerman, Iran; 3 Cellular and Molecular Research Center, Gerash University of Medical Sciences, Gerash, Iran

**Keywords:** Childhood acute lymphoblastic Leukemia, *XRCC1*, *NBN*, Single nucleotide variants, High-resolution melting analysis

## Abstract

**Objective:**

Acute lymphoblastic leukemia (ALL) is one of the most common cancers in children for which the exact pathogenesis is not yet known. Single-nucleotide variants (SNVs) in different DNA repair genes are reported to be associated with ALL risk. This study aimed to determine the association between *XRCC1* (rs1799782) and *NBN* (rs1805794, rs709816) SNVs and childhood ALL risk in a sample of the Iranian population. Fifty children with ALL and 50 age- and sex-matched healthy children were included in this case–control study. Genotyping of the mentioned SNVs was done by high-resolution melting (HRM) analysis.

**Results:**

The prevalence of all three SNVs in *XRCC1* and *NBN* genes did not differ between the patient and control groups, and these polymorphisms were not associated with childhood ALL risk (*P* > 0.05). HRM was a practical method for the detection of SNVs in *XRCC1* and *NBN* genes. We found no significant association between *XRCC1* (rs1799782) and *NBN* (rs1805794, rs709816) SNVs and childhood ALL risk.

**Supplementary Information:**

The online version contains supplementary material available at 10.1186/s13104-022-05918-3.

## Introduction

Globally, acute lymphoblastic leukemia (ALL) is the first most common form of childhood cancer and constitutes about 25–30% of all malignancies in those aged 2–5 years [1]. ALL is a heterogeneous disorder that arises from a malignant transformation and proliferation of lymphoid progenitors in bone marrow, peripheral blood, lymph nodes, and thymus [2]. It has been hypothesized that the initiation of ALL occurs in early infancy [3]. Overall survival (OS) in children is estimated close to 90% but patients with relapsed/refractory ALL present a therapeutic challenge [1, 4]. ALL progresses rapidly and it is suggested that a combination of genetic susceptibility and environmental factors contribute to disease development. However, the exact roles of both genetic and environmental factors as risk factors for ALL remain poorly understood and they require further study [5–7]. One of the major guilty of ALL pathogenesis is DNA damage. Humans are routinely adventured to endogenous and exogenous damaging factors [8]. Environmental exposures such as ultraviolet light, ionizing radiation, and chemical agents cause exogenous DNA damage. In addition, DNA may be damaged by endogenous factors such as oxidative stress, alkylation, hydrolysis (deamination, depurination, and depyrimidination), and mismatch of DNA bases [8, 9]. To overcome these lesions and maintain genome integrity, cells possess several DNA repair systems that remove many DNA damages [10]. These mechanisms are double-strand break repair, direct repair (MGMT), base excision repair (BER), nucleotide excision repair, and mismatch repair pathway [11]. In several studies have been reported that single nucleotide variants (SNVs) in genes encoding DNA repair proteins could be associated with ALL risk [12].

The BER pathway is primarily responsible for removing DNA lesions such as oxidized bases, alkylated bases, and abasic (AP) sites [13]. This pathway is a multistep process that requires the activation of several proteins and the deficiencies and changes in the efficiency of these proteins have been associated with diminished DNA repair capacity and individual susceptibility to cancer [14]. The x-ray repair cross-complementing group 1 (*XRCC1*) is one of the BER components. *XRCC1* gene is located on the long arm of chromosome 19 (19q13.2) and consists of 17 exons encoding a 633 amino acid protein which plays a crucial role as a scaffold and multifunctional protein in BER [9]. *XRCC1* has a domain for biologically significant interactions with other BER proteins, including poly (ADP-ribose) polymerase 1 (PARP-1), the gap-filling DNA polymerase β (POLβ), the DNA 30- phosphatase (PNKP), and DNA ligase 3a (LIG3a) [15]. SNVs are DNA base variants with a frequency of more than 1% in the human population [16]. SNV in the coding sequences of *XRCC1* was shown to potentially associate with modulated DNA repair capacity, making it a risk factor to occurrence of various cancers, especially hematopoietic diseases [17]. Three common SNVs have been identified in the *XRCC1* gene in humans (Arg194Trp, Arg280His, and Arg399Gln) [18, 19]. One of them is rs1799782 (at position 26304 on exon 6, base G to A, amino acid Arg to Trp) in the codon 194 which may alter *XRCC1* function [20].

Another DNA repair protein is Nibrin, encoded by the *NBN* gene located on chromosome 8q21. This protein is associated with both homologous recombination (HR) and non-homologous end-joining (NHEJ) DNA double-strand breaks (DSBs) repair pathways [21–23]. The protein is a member of the NBS1/hMre11/RAD50 (N/M/R) complex that plays a key role in DNA DSBs repair [24–26]. Genetic variants in *NBN* may modulate the function of the protein and so the DNA repair capacity. In the past decade, 675 SNVs were reported for the *NBN* gene to be associated with multiple cancers [27]. Two common *NBN* SNVs, based on minor allele frequency of > 5%, are rs1805794 in the codon 185 (exon 8, base C to G, amino acid Glu to Gln) and rs709816 in the codon 399 (exon 8, base A to G, Asp to Asp) which may alter *NBN* function [28].

According to this fact that the prevalence of genetic polymorphisms is racially dependent, so evaluation of these variations in different populations provides valuable information about leukemia susceptibility and disease prognosis. To enhance our knowledge about these variations, we designed a case–control study to investigate the association between *XRCC1* (rs1799782) and *NBN* (rs1805794, rs709816) genes polymorphisms and ALL risk in the Iranian population (Kerman province).

## Main Text

### Methods

#### Participants

This case–control study included 50 children diagnosed with ALL (based on current 2008 World Health Organization criteria) and 50 healthy children with no previous history of malignancy. All subjects in both case and control groups were matched for age and sex.

#### DNA extraction

Genomic DNA was extracted from 1.5 mL EDTA-anticoagulated whole blood samples using the modified salting-out method [29]. Extracted DNA samples were stored at − 20 °C until genotyping.

#### SNV genotyping by high-resolution melting (HRM) analysis

HRM analysis was performed using the Rotor-Gene^®^ Q instrument (QIAGEN’s real-time PCR cycler). According to the manufacturer’s protocol of 5 × HOT FIREPol^®^ EvaGreen^®^ HRM Mix (Solis BioDyne, Estonia), Components of an HRM reaction included: 1 × HRM Master Mix, 100 nM forward primer (10 pmol/μL), 100 nM reverse primer (10 pmol/μL), 50 ng/μL DNA template and H2O PCR grade. The final volume of the reaction mixture was 20 μL. Then, DNA was amplified under these cycling conditions: Initial activation at 95 °C for 12 min, followed by 40 cycles of amplification consisting of denaturation at 95 °C for 15 s, annealing at 67 °C for 20 s, and extension at 72 °C for 20 s. Finally, an HRM step was performed with an increase in temperature from 65 to 90 °C at a ramp rate of 0.1 °C per second. All data were analyzed using Rotor-Gene Q Series Software. Primer sequences and general information for each SNV are shown in Additional file 1: Table S1.

#### DNA sequencing

Because of obtaining different groups of melt curves and to ensure the accuracy of genotyping by HRM assay, three samples from each group were sent to BIONEER Corporation (South Korea) for direct DNA sequencing.

#### Statistical analysis

Pearson Chi-square test was used to compare the frequency of genotypes and alleles between case and control groups. To investigate the expected frequency of control genotypes, Hardy–Weinberg equilibrium (HWE) was applied. To determine the association of each genotype and risk of ALL, odds ratios (ORs) and 95% confidence intervals (CIs) were calculated using multivariate logistic regression. Statistical analyses were carried out with SPSS software version 23.0. A *P* value less than 0.05 was considered statistically significant.

### Results

#### Baseline characteristics of patients and controls

Our study included 50 children with ALL (age 5.5 ± 5.4 years) and 50 healthy children (age 6.2 ± 2.8 years). Demographic and hematologic characteristics of participants are presented in Table 1. All subjects in both case and control groups were matched for age (P = 0.41) and sex (P = 0.83). Hematologic parameters including red blood cell count, hemoglobin, hematocrit and platelet count were significantly associated with childhood ALL (P < 0.0001).

**Table 1:** Demographic and hematologic parameters

Parameters		Male	Female	P-value
Gender				
Control	Count	16 (32.0%)	34 (68.8%)	0.832
Patient	Count	17 (34.0%)	33 (66.0%)	

	N	Mean ± SD	Median	IQR	P-value
Age					
Control	50	6.2 ± 2.8	6	5.25	0.418
Patient	50	5.5 ± 5.4	5	4.5	
Red blood cells count × 10^12^/L					
Control	50	4.62 ± 0.55	4.67	0.79	< 0.0001
Patient	50	3.22 ± 1.07	3.47	1.55	
Hemoglobin (g/dL)					
Control	50	13.17 ± 0.88	13.2	1.33	< 0.0001
Patient	50	8.48 ± 2.69	9.05	4.02	
Hematocrit%					
Control	50	38.29 ± 2.70	38.7	4.65	< 0.0001
Patient	50	31.21 ± 7.46	31.79	12.35	
White blood cells count × 10^9^/L					
Control	50	7.69 ± 1.18	7.77	1.99	0.145
Patient	50	19.34 ± 30.30	6.3	14.58	
Platelet count × 10^9^/L					
Control	50	277.50 ± 277.68	217	108	< 0.0001
Patient	50	128.54 ± 157.54	54.5	198.25	

#### HRM and sequencing results

We used HRM assay to detect *XRCC1* (rs1799782 G > A) and *NBN* (rs1805794 C > G, rs709816 A > G) gene polymorphisms. After analyzing, it was found that the melting curves show different patterns, so several samples from each group were selected for sequencing. Supplementary figures (Additional file 2: Fig. S1, Additional file 3: Fig. S2, Additional file 4: Fig. S3) show the results of sequencing for each SNV as well as the genotyping results obtained by the HRM technique presented in three models (melting curve, normalized graph, and difference graph).

#### Genotype and allele frequency of XRCC1 and NBN genes polymorphisms and the association with childhood ALL

The genotype distributions for SNV rs1799782 (*XRCC1* A > G) and SNV rs709816 (*NBN* G > A) were in Hardy–Weinberg Equilibrium (*P* > 0.05) in both patients and controls, but for SNV rs1805794 (*NBN* C > G) were not (*P* < 0.001) in both groups (Table 2 caption). The frequency of genotypes and alleles in the studied groups is presented in Table 2. Our data revealed no significant differences in genotype and allelic distribution of all three SNVs between the patient and control groups (*P* > 0.05). Accordingly, we could not find any statistically significant association between these SNVs, rs1799782 (*XRCC1* G > A; OR = 0.57, 95% CI 0.13–2.54, *P* = 0.46, AA vs. GG; OR = 0.57, 95% CI 0.13–2.54, *P* = 0.46, Dominant model, GA + AA vs. GG; OR = 1.74, 95% CI 0.39–7.17, *P* = 0.46, Recessive model, GG + GA vs. AA; OR = 0.57, 95% CI 0.20–1.64, *P* = 0.29, A allele vs. G allele), rs1805794 (*NBN* C > G; OR = 1.37, 95% CI 0.62–3.02, *P* = 0.42, CG vs. CC; OR = 1.37, 95% CI 0.62–3.02, *P* = 0.42, Dominant model, CG + GG vs. CC; OR = 1.24, 95% CI 0.65–2.38, *P* = 0.5, G allele vs C allele) and rs709816 (*NBN* A > G; OR = 0.96, 95% CI 0.41–2.23, *P* = 0.96, AG vs. AA; OR = 1.68, 95% CI 0.47–6.00, *P* = 0.42, GG vs. AA; OR = 1.08, 95% CI 0.48–2.21, *P* = 0.83, Dominant model, AG + GG vs. AA; OR = 0.58, 95% CI 0.17–1.92, *P* = 0.37, Recessive model, AA + AG vs. GG; OR = 1.19, 95% CI 0.66–2.12, *P* = 0.55, G allele vs. A allele), and the risk of childhood ALL.

**Table 2:** Genotype and allele frequency of *XRCC1* and *NBN* genes polymorphisms and the association with childhood ALL

	Group		P-Value	OR	95% CI for OR	
	Control	Patient			Lower	Upper
*XRCC1* rs1799782 G > A						
Genotype						
GG	45 (48.9%)	47 (51.1%)	–	1	–	–
GA	0 (0.0%)	0 (0.0%)	–	–	–	–
AA	5 (62.5%)	3 (37.5%)	0.465	0.574	0.130	2.545
Dominant model						
GA + AA	5 (62.5%	3 (37.5%)	0.465	0.574	0.130	2.545
Recessive model						
GG + GA	45 (48.9%)	47 (51.1%)	0.465	1.74	0.393	7.713
Allele						
G	90 (48.9%)	94 (51.1%)	–	1	–	–
A	10 (62.5%)	6 (37.5%)	0.297	0.574	0.201	1.646
*NBN* rs709816 A > G						
Genotype						
AA	21 (51.2%)	20 (48.8)	–	1	–	–
AG	24 (52.2%)	22 (47.8%)	0.963	0.962	0.415	2.235
GG	5 (38.5%)	8 (61.5%)	0.420	1.68	0.470	6.007
Dominant model						
AG + GG	29 (49.2%)	30 (50.8%)	0.839	1.086	0.489	2.214
Recessive model						
AA + AG	45 (51.7%)	42 (48.3%)	0.376	0.583	0.177	1.925
Allele						
A	66 (51.5%)	62 (48.4%)	–	1	–	–
G	34 (47.2%)	38 (52.7%)	0.556	1.190	0.667	2.121
*NBN* rs1805794 C > G						
Genotype						
CC	28 (53.8%)	24 (46.2%)	–	1	–	–
CG	22 (45.8%)	26 (54.2%)	0.42	1.378	0.628	3.029
GG	0 (0.0%)	0 (0.0%)	–	–	–	–
Dominant model						
CG + GG	22 (45.8%)	26 (54.2%)	0.423	1.378	0.628	3.029
Recessive model						
CC + CG	50 (50.0%)	50 (50.0%)	–	–	–	–
Allele						
C	78 (51.3%)	74 (48.6)	–	1	–	–
G	22 (45.8%)	26 (54.1%)	0.508	1.246	0.650	2.388

*NBN* SNVs interaction					
	B	P-Value	OR	95% CI for OR	
				Lower	Upper
rs709816 by rs1805794	1.119	0.336	3.062	0.313	29.948

Hardy–Weinberg Equilibrium (HWE) results for *XRCC1* and *NBN* genes polymorphisms: rs1799782 (XRCC1 G > A), Control Group (χ2 = 0.13, P = 0.7), Case Group (χ2 = 0.04, P = 0.8); rs1805794 (NBN C > G): Control Group (χ2 = 3.97, P = 0.04), Case Group (χ2 = 6.17, P = 0.01); rs709816 (NBN A > G): Control Group (χ2 = 0.24, P = 0.62), Case Group (χ2 = 0.21, P = 0.63)

In addition, the interaction analysis of the *NBN* SNVs (rs709816 by rs1805794) did not showed any statistically significant association with childhood ALL (OR = 3.06, 95% CI 0.31–29.94, P = 0.33). Multivariate regression analysis results, expressing the impact of each variable independently, are presented in Table 3. Accordingly, the studied SNVs were not associated with childhood ALL (P > 0.05).

**Table 3:** Multivariate regression analysis results

Variables	B	P-Value	Adjusted OR	95% CI for OR	
				Lower	Upper
rs1799782 (AA)	3.894	0.143	49.106	0.267	9047.383
rs1799782 (A allele)	− 0.585	0.278	0.557	0.194	1.603
rs1805794 (CG)	0.325	0.863	1.384	0.034	55.566
rs1805794 (G allele)	0.328	0.353	1.388	0.695	2.773
rs709816 (AG)	0.158	0.932	1.171	0.031	44.399
rs709816 (GG)	− 0.821	0.765	0.44	0.002	95.909
rs709816 (G allele)	0.285	0.364	1.33	0.718	2.462
Red blood cell count	0.226	0.866	1.254	0.091	17.271
Hemoglobin	− 3.159	0.012	0.042	0.004	0.497
Hematocrit	− 0.485	0.033	0.616	0.395	0.961
Platelet count	− 0.002	0.705	0.998	0.987	1.009

### Discussion

ALL is a complex disease influenced by both genetic and environmental factors [30]. One of the important factors in the development of this leukemia is genetic variations including SNVs as the most common type of variations [30]. In the present study, we investigated the influence of polymorphisms within *XRCC1* (rs1799782 G > A) and *NBN* (rs1805794 C > G, rs709816 A > G) genes on susceptibility to childhood ALL in a sample of the Iranian population by HRM analysis as a powerful, cost-effective and practicable method [31, 32] for detecting SNVs and discriminating the wild type and mutant genotypes with melting temperature shifts more than 0.4 °C [33]. rs1799782 G > A is located in the coding sequence of *XRCC1* and associated with modulated DNA repair capacity [34]. According to the present results, there was no significant association between this SNV and ALL risk in our population. Similar to our finding, Joseph et al. showed that this polymorphism did not affect the susceptibility to ALL in Indian children [35]. Besides, no significant differences were observed among Turkish patients with childhood ALL and the controls concerning this polymorphism. In this study, however, Batar et al. reported that the combined *XRCC1* AG/AA variant genotypes were associated with increased risk for ALL in females (OR = 5.47; 95% CI 1.49–20.10; p = 0.008) [34]. A meta-analysis of 38 case–control studies indicated that *XRCC1* Arg194Trp may be a biomarker of cancer susceptibility [36]. Other SNVs examined in the present study were rs1805794 C > G and rs709816 A > G, localized to the coding sequences of the *NBN* gene, which may modulate the protein function and so the DNA DSB repair capacity [37]. In our study, no significant association was observed between the *NBN* polymorphisms and susceptibility to childhood ALL. Similar to our results, Mosor et al. genotyped six polymorphisms of the *NBN* gene in the Poland population and suggested that one specific variant of the gene may be associated with childhood acute leukemia but no significant association was detected for the remaining five *NBN* polymorphisms including rs1805794 and rs709816 [38]. Consistently, in the other study on 460 pediatric ALL cases and 552 healthy controls in the European population (Germany), Smolkova et al. also did not show any association between the *NBN* polymorphisms and ALL risk [39]. These results show that our population, at least in part, is closely related to the European population. In contrast, in the study of the Chinese population conducted on 175 patients with ALL and 350 controls, Jiang et al. observed a significant difference in frequencies of rs1805794 C/G between cases and controls (*P* < 0.0001) and reported for the first time that CC genotype carriers were associated with a 3.4-fold elevated risk of ALL, compared with the non-carries [27]. Also, a meta-analysis of rs1805794 including 3065 subjects revealed the association of CC genotype with approximately 1.70-fold increased risk of acute leukemia. In contrast, there was no association between rs709816 and risk of acute leukemia in the meta-analysis of including 1,485 samples [37].

In conclusion, we cannot recognize any association between the *XRCC1* and *NBN* polymorphisms and susceptibility to childhood ALL in a sample of the Iranian population.

## Limitations

- The discrepancy of our results with other studies may be due to the small sample size and so we suggest that similar studies be conducted on the larger sample size and different populations.
- The three SNVs in DNA repair genes may not give as comprehensive a view of genetic variation as genomic sequencing does.
- These SNVs may probably exert their effects through complex gene–gene and/or gene-environment interactions. Such interactions were not investigated in this study.

## Supplementary Information

**Additional file 1: Table S1.** Primer sequences for detection of *XRCC1* and *NBN* gene polymorphisms using the HRM method.

**Additional file 2: Fig. S1.** Genotyping of *XRCC1* (rs1799782) gene polymorphism by HRM analysis. (A) rs1799782 G>A Normalized graph; (B) rs1799782 G>A Melting curve; (C) rs1799782 G>A Difference graph; (D) rs1799782 G>A sequencing results.

**Additional file 3: Fig. S2.** Genotyping of *NBN* (rs1805794) gene polymorphism by HRM analysis. (A) rs1805794 C>G Normalized graph; (B) rs1805794 C>G Melting curve; (C) rs1805794 C>G Difference graph; (D) rs1805794 C>G sequencing results.

**Additional file 4: Fig. S3.** Genotyping of *NBN* (rs709816) gene polymorphism by HRM analysis. (A) rs709816 A>G Normalized graph; (B) rs709816 A>G Melting curve; (C) rs709816 A>G Difference graph; (D) rs709816 A>G sequencing results.

## Data Availability

The datasets used and/or analysed during the current study are not publicly available due to the possibility of compromising the privacy of individuals. According to the written approval forms accepted by the Ethics Committee of the Kerman University of Medical Sciences (KMU), the data will only be accessible to researchers within the project. The data would be available from the corresponding author on reasonable request.

## Abbreviations

ALL: Acute lymphoblastic leukemia
BER: Base excision repair
CI: Confidence intervals
DSBs: DNA double-strand breaks
HRM: High-resolution melting
HR: Homologous recombination
LIG3a: Ligase 3a
NHEJ: Non-homologous end-joining
OS: Overall survival
ORs: Odds ratios
PARP-1: Poly (ADP-ribose) polymerase 1
POLβ: Polymerase β
*XRCC1*: X-ray repair cross-complementing group 1
SNVs: Single nucleotide variants
