# Supplementary material for: Association of DNA repair genes polymorphisms with childhood acute lymphoblastic leukemia: a high-resolution melting analysis
Source: BMC Res Notes. 2022 Feb 14;15:46. doi: 10.1186/s13104-022-05918-3 (PMC8842869; doi:10.1186/s13104-022-05918-3)
Supplement: Supplementary file 1 — Additional file 1: Table S1. Primer sequences for detection of XRCC1 and NBN gene polymorphisms using the HRM method. [file 13104_2022_5918_MOESM1_ESM.docx]

**Additional file 1: Table S1**

Primer sequences for detection of *XRCC1* and *NBN* gene polymorphisms using HRM method

| SNP (location) | Primer sequences | SNP sequence | Amplicon Size (bp) |
| --- | --- | --- | --- |
| **XRCC1**  **rs1799782** | F: 5´ GCTCACCTGGGGATGTCTTG 3´  R: 5´ GTTCCGTGTGAAGGAGGAGG 3´ | G$>A$ | 97 |
| **NBN**  **rs1805794** | F: 5´ TACCTTTCAATTTGTGGAGGCTG 3´  R: 5´ GTGCACTCATTTGTGGACG 3´ | C$>G$ | 100 |
| NBN  rs709816 | F: 5´ GCAGGACTCCTTTACAGTGGG 3  R: 5´ ACAGGGATTTGAGTGAAAGGC 3´ | A$\boldsymbol{>G}$ | 101 |
