# Supplementary figures and images for: Association of DNA repair genes polymorphisms with childhood acute lymphoblastic leukemia: a high-resolution melting analysis
Source: BMC Res Notes. 2022 Feb 14;15:46. doi: 10.1186/s13104-022-05918-3 (PMC8842869; doi:10.1186/s13104-022-05918-3)

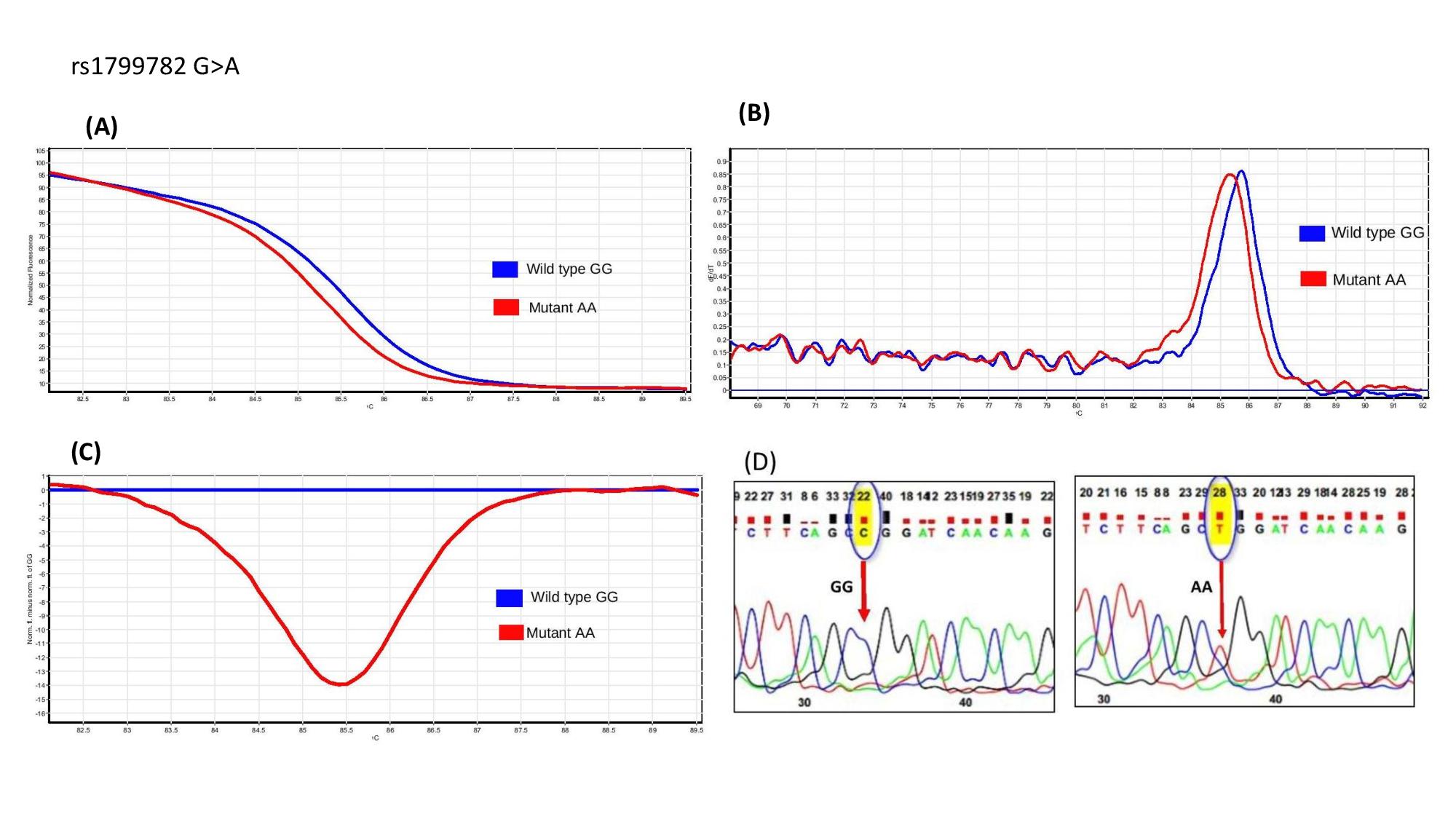

Supplement: Supplementary file 2 — Additional file 2: Fig. S1. Genotyping of XRCC1 (rs1799782) gene polymorphism by HRM analysis. (A) rs1799782 G>A Normalized graph; (B) rs1799782 G>A Melting curve; (C) rs1799782 G>A Difference graph; (D) rs1799782 G>A sequencing results. [file 13104_2022_5918_MOESM2_ESM.jpg]

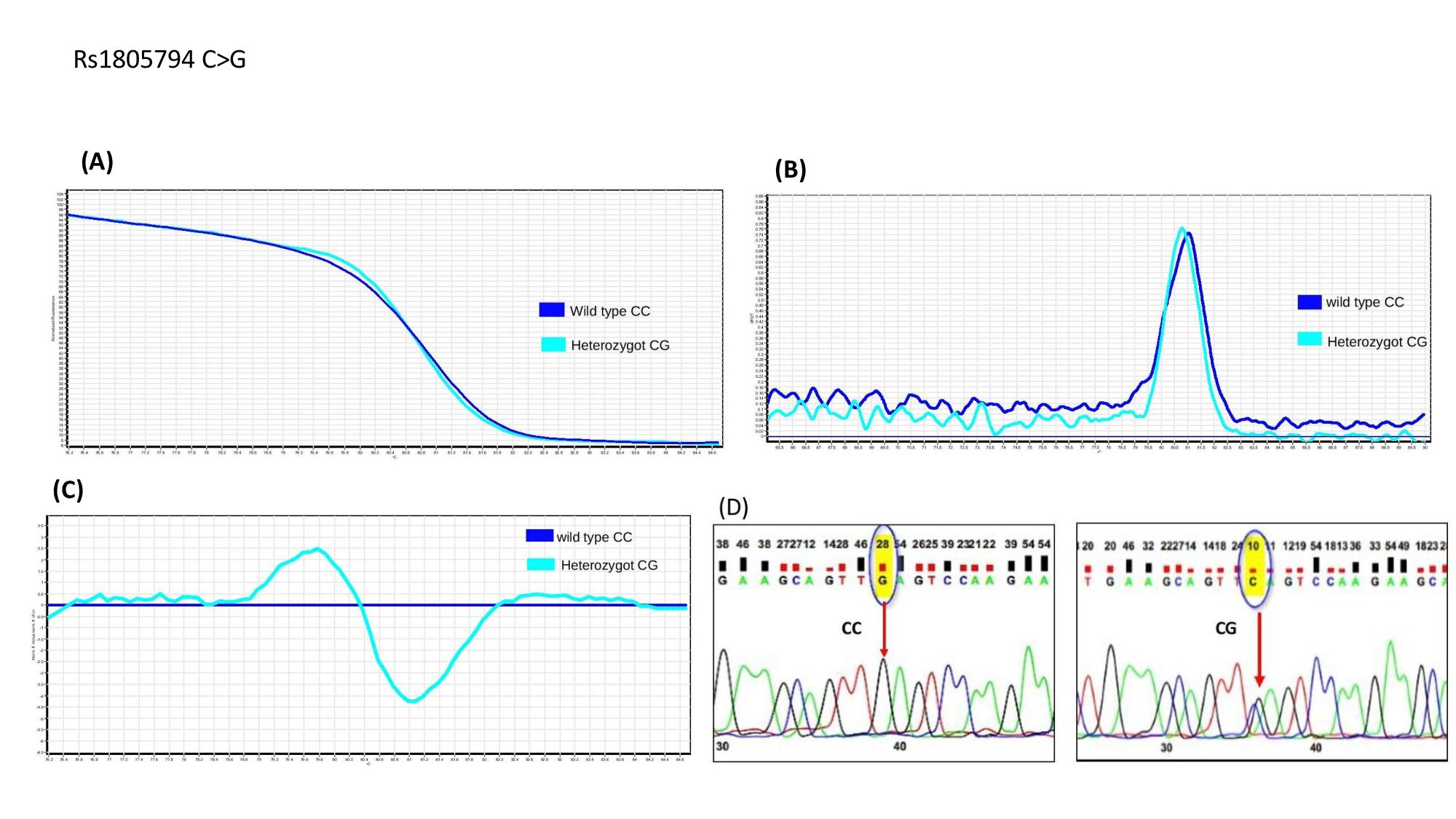

Supplement: Supplementary file 3 — Additional file 3: Fig. S2. Genotyping of NBN (rs1805794) gene polymorphism by HRM analysis. (A) rs1805794 C>G Normalized graph; (B) rs1805794 C>G Melting curve; (C) rs1805794 C>G Difference graph; (D) rs1805794 C>G sequencing results. [file 13104_2022_5918_MOESM3_ESM.jpg]

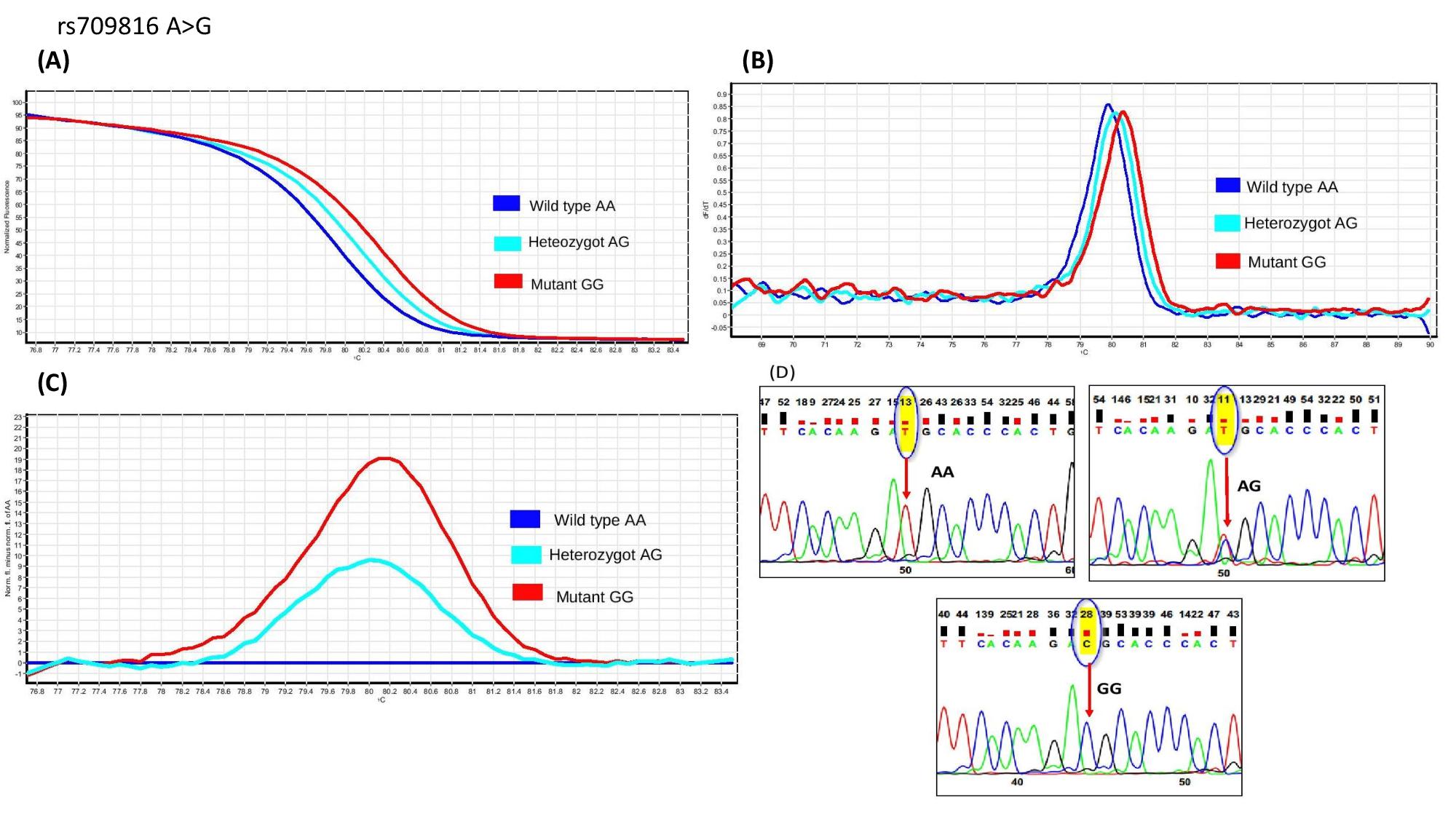

Supplement: Supplementary file 4 — Additional file 4: Fig. S3. Genotyping of NBN (rs709816) gene polymorphism by HRM analysis. (A) rs709816 A>G Normalized graph; (B) rs709816 A>G Melting curve; (C) rs709816 A>G Difference graph; (D) rs709816 A>G sequencing results. [file 13104_2022_5918_MOESM4_ESM.jpg]
